# Higher cytolytic score correlates with an immunosuppressive tumor microenvironment and reduced survival in glioblastoma

**DOI:** 10.1038/s41598-020-73793-8

**Published:** 2020-10-16

**Authors:** Alexander F. Haddad, Jia-Shu Chen, Taemin Oh, Matheus P. Pereira, Rushikesh S. Joshi, Manish K. Aghi

**Affiliations:** 1 School of Medicine, University of California San Francisco, 513 Parnassus Ave., San Francisco, CA 94143-0410 USA; 2 Department of Neurological Surgery, University of California San Francisco, San Francisco, USA; 3 School of Medicine, University of California San Diego, San Diego, USA

**Keywords:** Cancer microenvironment, Tumour immunology, Neurology, Oncology

## Abstract

Cytolytic score (CYT), calculated from mRNA expression levels of granzyme and perforin, positively correlates with CD8+ T cell infiltration/activity in a variety of cancers. Unlike other cancers, higher CYT has been associated with worse prognosis in glioblastoma (GBM). To address this discrepancy, we sought to investigate the relationship between CYT and immune checkpoint gene score (ICGscore), as well as their correlation with patient survival and tumor immune cell infiltration. Clinical and RNA-sequencing data for patients with newly diagnosed GBM were obtained from The Cancer Genome Atlas. Maximally-selected rank statistics was used to dichotomize subgroups. CIBERSORT was used to estimate abudence of immune cell-types. Spearman correlation was used to characterize the relationship between CYT and ICGscore. Kaplan–Meier curves were generated for survival analysis. Overall, 28/151 patients had high CYT. High CYT was associated with a mesenchymal subtype (p < 0.001) and worse survival (7.45 vs. 12.2 months, p < 0.001). There were no differences in patient demographics, IDH/MGMT mutation status, or treatment. On subgroup analysis, patients with high CYT/ICGscore had significantly increased CD8+ infiltration (p < 0.001), as expected, and worse survival (HR 0.445, p < 0.01). Furthermore, CYT strongly correlated with ICGscore (R_S_ = 0.675, p < 0.001). The high CYT/ICGscore subgroup was associated with greater infiltration of M2 macrophages (p = 0.011) and neutrophils (p = 0.055). Our study highlights a multidimensional immunosuppressive GBM microenvironment in patients with higher CYT and potentially identifies patients with high CYT/ICGscore as a subgroup that may particularly benefit from multi-faceted immunotherapies, given their already elevated tumor CD8+ T cell levels.

## Introduction

It is well-known that tumor cells can be antigenic due to abnormal protein products and expression^1,2^. A substantial body of literature has already established the prognostic benefit associated with increased tumor-infiltrating lymphocytes (TILs), especially cytotoxic T-cells, which can induce death of cancer cells through release of granzyme and perforin^3^. Indeed, high levels of TILs have been correlated with a survival benefit in a variety of cancers^4–7^. However, in glioblastoma (GBM), which is a notoriously immunosuppressive and treatment-resistant neoplasm in the immunoprivileged microenvironment of the brain, the relationship between survival and TILs remains controversial, with conflicting studies existing in the literature^8–11^.

The cytolytic activity score (CYT) was developed by Rooney et al. as a quantitative means of assessing cytotoxic T cell infiltration and activity and is calculated using transcript levels of effector proteins granzyme (GZMA) and perforin (PRF1)^7^. Overall, it is believed that higher CYT has a positive correlation with prolonged survival in a variety of cancers^7^. In colorectal and pancreatic cancers, for example, prior work has shown a positive correlation between high CYT, immune checkpoint gene (ICG) expression, and prolonged survival^4–6^. While a high CYT has similarly been shown to correlate with higher expression of the immunosuppressive PD1/PDL1 axis in GBM, it has also been associated with worse overall survival, in contrast to other cancers^10^. The specific mechanisms underlying this discrepancy are yet unclear. The current study represents an effort to better understand the relationship between GBM and the immune response that might potentially be driving this effect, including factors such as CYT, ICG expression, and immune cell infiltration. As it is unlikely that any single variable is responsible, we sought to shed light on these relationships by leveraging the comprehensive wealth of RNA sequencing data from GBM patients provided in The Cancer Genome Atlas (TCGA).

## Materials and methods

### Acquisition and processing of genomic and clinical data

Clinical and RNA sequencing data for 151 patients diagnosed with primary glioblastoma were acquired from TCGA via cBioPortal (https://www.cbioportal.org/) and the Broad Institute’s Firehose Pipeline (https://gdac.broadinstitute.org/), respectively. Patients without gene expression data or survival outcome data were excluded from further analysis. Out of the 152 TCGA patients with available RNA sequencing data, one patient was excluded due to a lack of survival data. A similar dataset of GBM patients was also acquired from the Chinese Glioma Genome Atlas (CGGA, https://www.cgga.org.cn/index.jsp) and used to validate TCGA findings. Pre-processing and statistical analysis were performed in RStudio (RStudio, Inc., Version 1.2.1335).

Gene expression data was first converted to transcripts per million (TPM) by multiplying the scaled and estimated frequency of a gene’s transcripts amongst the total number of sequenced transcripts by 1E6. CYT was derived for each patient sample by finding the geometric mean of GZMA and PRF1 expression using previously established methodology^7^. To dichotomize the population into low vs high CYT, maximally selected rank statistics was used to determine the score threshold that provided the most significant difference in overall survival.

Expression thresholds for six ICGs (PD1, PDL1, CTLA4, TIM3, LAG3, and IDO) were also determined using maximally selected rank statistics in order to classify each patient sample as high vs low expression for each of the aforementioned genes. An ICG score was determined by calculating the geometric mean of the six ICG gene expression values for each patient. Patients were then categorized into one of four groups, depending on their CYT and ICG scores: High CYT/High ICGscore, High CYT/Low ICGscore, Low CYT/High ICGscore, Low CYT/Low ICGscore.

### Statistics

Kaplan–Meier survival analysis with log-rank test was performed to compare differences in overall survival. Univariate Cox regression was performed for each clinical variable and subgroup classification in order to determine factors associated with overall survival, as defined by the time between date of diagnosis to date of death. All statistically significant variables (p < 0.05) were then included in a multivariate Cox regression model to identify the covariates most capable of predicting patient outcome. Hazard ratios, p values, and 95% confidence intervals were obtained for each covariate.

The CIBERSORT deconvolution algorithm was used to estimate the intratumor composition of 22 immune cell types in subgroups of interest. The Mann–Whitney U test was used for analyzing continuous variables, including immune cell composition and quantitative clinicopathological features. The Pearson’s chi-squared test was used to determine differences in categorical or discrete variables, including sex, ethnicity, clinical treatment, tumor subtype, IDH mutation classification, and MGMT methylation status. Spearman correlation and linear regression were used to characterize the relationship between CYT and ICG expression. A two-sided p value < 0.05 was used as the threshold for statistical significance in all analyses.

## Results

### High CYT is associated with reduced survival

**Figure 1:**
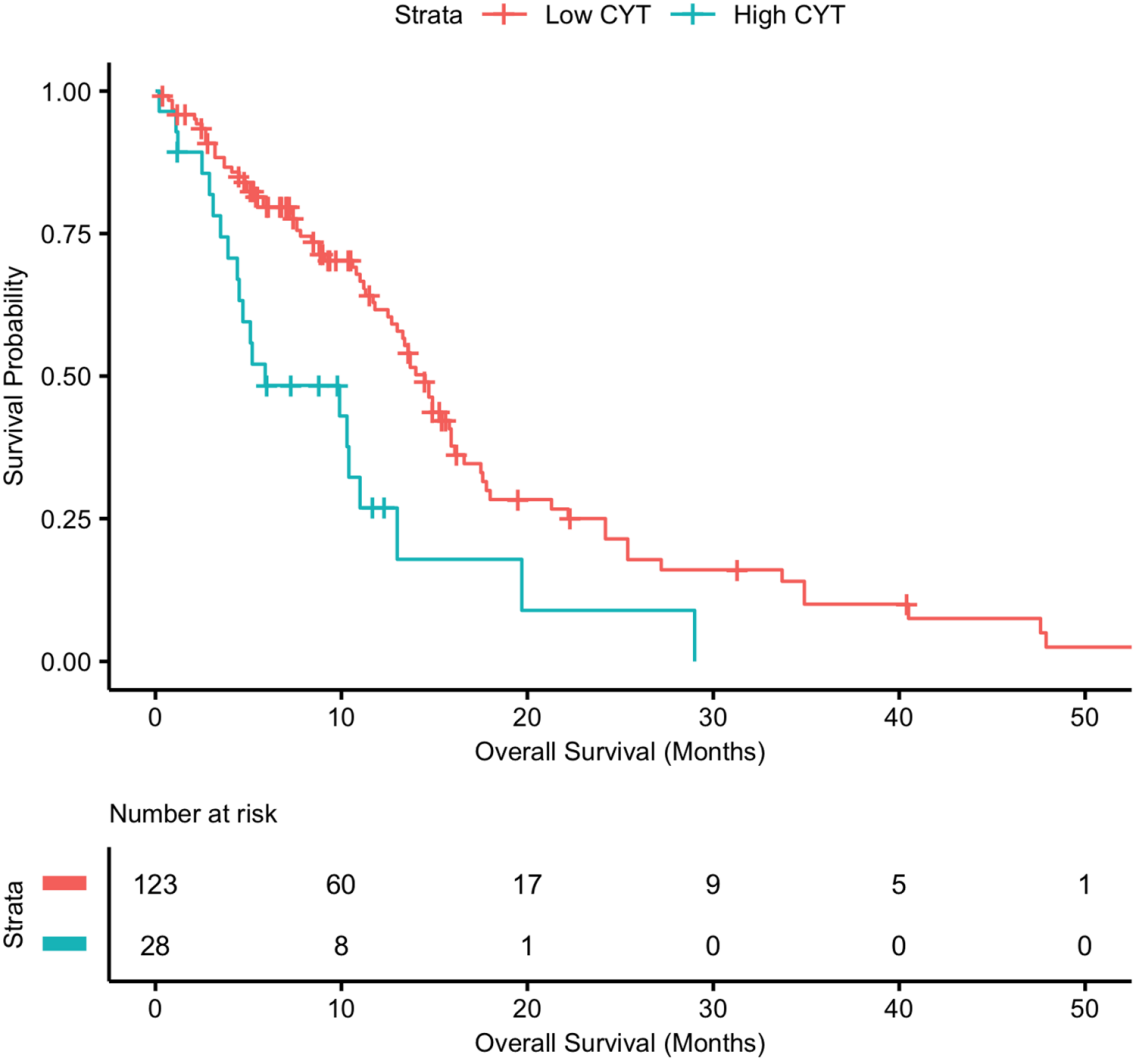
Survival analysis of GBM patients with high vs. low CYT. High CYT was determined using maximally selected rank statistics. Patients with high CYT had reduced survival when compared to those with low CYT (7.45 vs. 12.2 months, p < 0.001).

We first began by analyzing the relationship between CYT and patient survival. Of the 151 GBM patients, 28 had a high CYT, as defined using maximally selected rank statistics (> 5.52 TPM). The median survival of the patient cohort was 9.1 months. Compared to patients with low CYT, patients with high CYT were more likely to have a mesenchymal subtype (67.9% vs. 23.4%, p < 0.001). There were no other significant differences between high vs low CYT groups with regards to demographics, GBM subtype, IDH mutation, MGMT methylation status, or treatment received (Table 1). Patients with high CYT had worse overall survival compared to low CYT patients (7.45 vs. 12.2 months, p < 0.001) (Fig. 1). On multivariate analysis, higher CYT continued to be significantly associated with reduced survival (HR 2.87, 95% CI 1.55–5.32; p < 0.001) when accounting for other common predictors of survival such as GBM subtype (Table 2). Increased age (> 60) was also independently associated with reduced patient survival (HR 1.65, 95% CI 1.08–2.54; p = 0.021) (Table 2).

**Table 1:** Patient demographic and clinical information in high CYT and low CYT groups.

Variable	High CYT (n = 28)	Low CYT (n = 123)	*p* value
Age (mean years)	65.57 ± 10.50	59.32 ± 13.46	**0.039**
**Sex**			0.277
Female	13 (46.4%)	41 (33.3%)	
Male	15 (53.6%)	82 (66.7%)	
**Ethnicity**			0.699
African	2 (7.1%)	8 (6.5%)	
Asian	0	5 (4.1%)	
White	26 (92.9%)	109 (88.6%)	
**Tumor subtype **			**<0.001**
Classical	1 (3.6%)	38 (30.9%)	
Mesenchymal	19 (67.9%)	29 (23.6%)	
Neural	5 (17.9%)	21 (17.1%)	
Proneural	3 (10.7%)	25 (20.3%)	
**IDH mutation**			0.411
R132H	0	7 (5.7%)	
Wild type	28 (100%)	112 (91.1%)	
**MGMT methylation**			0.611
Methylated	11 (39.3%)	43 (35.0%)	
Unmethylated	13 (46.4%)	52 (42.3%)	
**Recurrence**			0.889
Disease-free	14 (50.0%)	60 (48.8%)	
Recurrence	14 (50.0%)	62 (50.4%)	
**Treatment**			0.639
Standard radiation	2 (7.1%)	9 (7.3%)	
Nonstandard radiation	1 (3.6%)	3 (2.4%)	
TMZ chemo	0	1 (0.8%)	
Standard radiation + TMZ chemo	7 (25.0%)	17 (13.8%)	

Bold corresponds to a *p* value < 0.05.

**Table 2:** Univariate and multivariate cox regression analysis of variables impacting GBM patient survival, including CYT.

Variables	HR (95% CI)	*p* value
**Univariate analysis (Cox regression)**		
Age (> 60 years)	1.684 (1.120–2.530)	**0.012**
Sex (male)	0.745 (0.496–1.119)	0.156
Tumor subtype		
Classical	0.735 (0.411–1.311)	0.297
G-CIMP	0.173 (0.040–0.743)	**0.018**
Mesenchymal	0.843 (0.476–1.493)	0.558
Neural	0.814 (0.442–1.499)	0.509
IDH1 (R132H)	0.240 (0.059–0.979)	**0.047**
MGMT (methylated)	0.625 (0.384–1.017)	0.058
CYT (high)	2.305 (1.403–3.786)	**< 0.001**
ICGscore (high)	1.354 (0.904–2.029)	0.141
**Multivariate analysis (Cox regression)**		
Age (> 60 years)	1.654 (1.079–2.535)	**0.021**
Tumor subtype		
Classical	0.707 (0.393–1.270)	0.245
G-CIMP	1.019 × 10^−7^ (0.0-Inf)	0.996
Mesenchymal	0.527 (0.274–1.013)	0.054
Neural	0.803 (0.436–1.479)	0.482
IDH1 (R132H)	2.221 × 10^6^ (0.0-Inf)	0.996
CYT (high)	2.874 (1.553–5.318)	**< 0.001**

Bold corresponds to a *p* value < 0.05.

### High CYT plus high ICG subgroup has the worst survival

**Figure 2:**
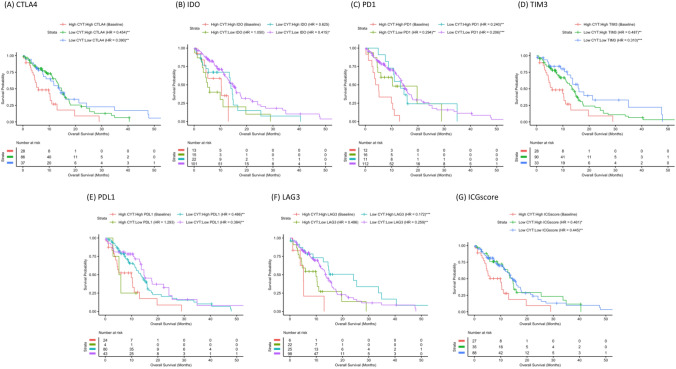
Survival analysis of CYT/ICG subgroups. Kaplan–Meir curve of CYT/ICG subgroups across all ICGs including (**A**) CTLA4, (**B**) IDO, (**C**) PD1, (**D**) TIM3, (**E**) PDL1, (**F**) LAG3 assessing their impact on patient survival. (**G**) Kaplan–Meir curve of the composite ICGscore subgroups is also displayed (HR 0.445, p < 0.01).

CYT and ICG subgroup analysis can be found in Fig. 2. We included six immune checkpoint genes that have been implicated in GBM immunosuppression: PDL1, PD1, CTLA4, IDO, TIM3, and LAG3^10,12–17^. Corresponding to what has been previously reported with CYT and the PD1/PDL1 axis in GBM^10^, we found that patients with high CYT and high expression of individual ICGs (HighCYT/HighICG) had significantly worse survival relative to other subgroups (Fig. 2A–F). To provide an overview of the relationship between CYT, overall ICG expression, and survival, we then evaluated a composite ICGscore, the geometric mean of the ICGs included in our study, in a similar manner to the individual genes analyzed. As expected, patients with HighCYT/HighICGscore had significantly worse survival than the LowCYT/LowICGscore (HR 0.445, p < 0.01) (Fig. 2G). There was one HighCYT/LowICGscore patient, limiting the analysis that could be performed in that subgroup.

### CYT positively correlates with ICGscore

**Figure 3:**
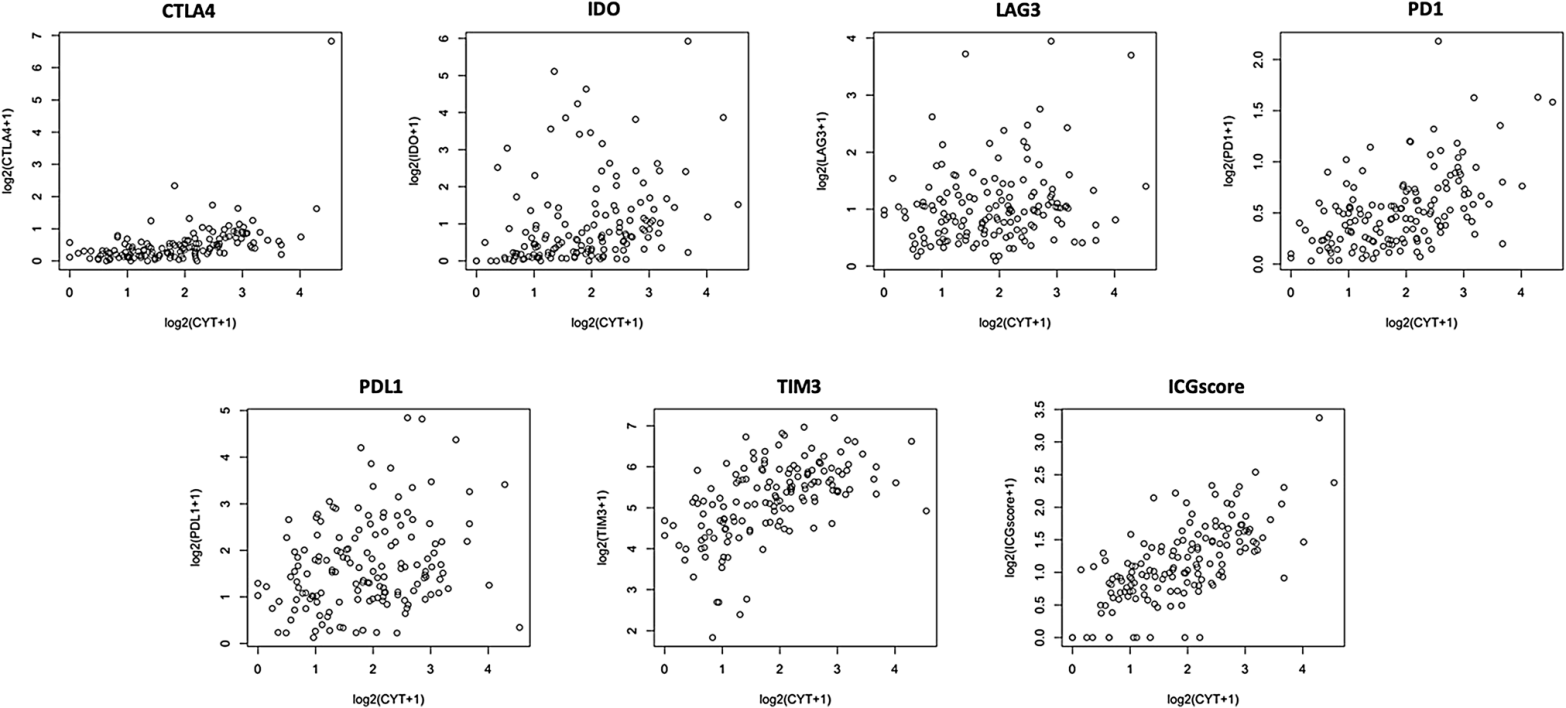
Spearman’s rank order correlation of ICG and CYT. Correlation between CYT and ICGs including CTLA4, IDO, LAG3, PD1, PDL1, and TIM3. The correlation between CYT and composite ICGscore is also highlighted (R_S_ = 0.675, p < 0.001).

CYT positively correlated with expression of all individual ICGs, including CTLA4 (R_S_ = 0.610, p < 0.001), IDO (R_S_ = 0.462, p < 0.001), LAG3 (R_S_ = 0.187, p = 0.02), PD1 (R_S_ = 0.500, p < 0.001), PDL1 (R_S_ = 0.255, p = 0.002), TIM3 (R_S_ = 0.594, p < 0.001) (Fig. 3). As expected, CYT also had a strong correlation with the overall ICGscore (R_S_ = 0.675, p < 0.001) (Fig. 3).

### HighCYT/HighICGscore correlates with a more immunosuppressive tumor microenvironment

**Figure 4:**
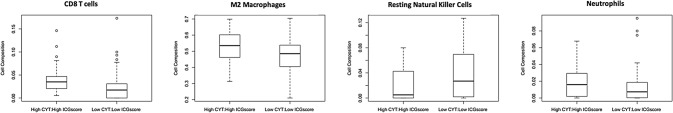
Immune cell infiltration between CYT/ICGscore subgroups. Immune cell populations differ between HighCYT/HighICGscore and LowCYT/LowICGscore subgroups. CD8+ T cells have increased infiltration in patients with HighCYT/HighICGscore (p < 0.001). M2 Macrophages are increased in patients with HighCYT/HighICGscore (p = 0.0107). Resting natural killer cells are lower in patients with HighCYT/HighICGscore (p = 0.0289). Neutrophils are increased in patients with HighCYT/HighICGscore, approaching a significant difference (p = 0.055).

We next sought to determine the role that the tumor microenvironment plays in the poor survival of HighCYT/HighICG patients by assessing differences in immune cell infiltration. We compared patients with HighCYT/HighICGscore to those with LowCYT/LowICGscore (Fig. 4). HighCYT/HighICGscore patients had significantly higher levels of CD8+ T cell tumor infiltration (p < 0.001) (Fig. 4) and reduced levels of resting NK cells (p = 0.03) (Fig. 4). Patients with HighCYT/HighICGscore also displayed higher levels of immunosuppressive immune cells, including a significantly higher level of M2 Macrophages (p = 0.011) (Fig. 4). Tumor-associated neutrophils, thought to be immunosuppressive^18^, were also higher in HighCYT/HighICGscore patients, trending towards significance (p = 0.055) (Fig. 4). None of the other 22 immune cell types assessed by CIBERSORT were significantly different between subgroups (p > 0.05).

### Validation of findings using the CGGA

**Figure 5:**
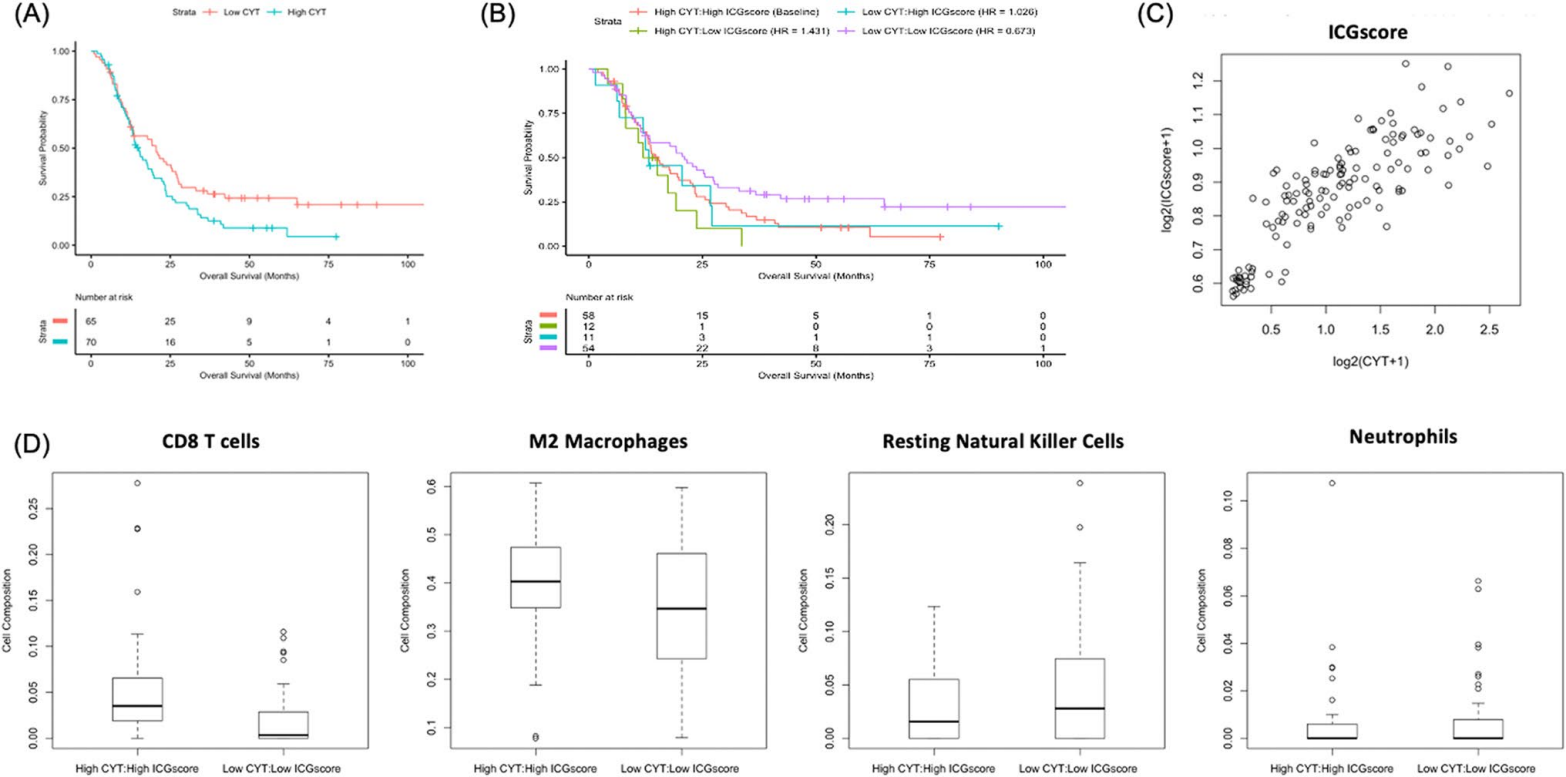
Validation study using CGGA GBM cohort. (**A**) Kaplan–Meier curve comparing high CYT patients with low CYT patients (19.1 vs. 25.7 months, p = 0.05). (**B**) Kaplan–Meier curve of CYT/composite ICGscore subgroups (HR = 0.673, p = 0.066). (**C**) Spearman’s rank order correlation between CYT and composite ICGscore (R_S_ = 0.8291, p < 0.001). (**D**) Comparison of immune cell infiltration between CYT/ICGscore subgroups. CD8 T-Cell (p < 0.001) and M2 Macrophage (p = 0.017) populations were both increased in patients with HighCYT/HighICGscore.

TCGA findings were validated using a cohort of 135 primary GBM patients from the CGGA, with a median survival of 14.1 months. Patients with high CYT had significantly reduced survival (19.1 vs. 25.7 months, p = 0.05) when compared to low CYT patients (Fig. 5A). In addition, when assessing CYT/ICGscore subgroups, LowCYT/LowICGscore patients had increased survival relative to HighCYT/HighICGscore patients, although this only approached significance (HR 0.673, p = 0.066, Fig. 5B). ICGscore was strongly correlated with CYT (R_S_ = 0.830, p < 0.001) (Fig. 5C). HighCYT/HighICGscore patients also had increased tumor infiltration of CD8 T cells (p < 0.001) and M2 Macrophages (p = 0.02) when compared to LowCYT/LowICGscore. There was no difference between groups with regards to tumor infiltration of resting NK cells (p = 0.176) or neutrophils (p = 0.699) (Fig. 5D).

## Discussion

Using RNA expression data from TCGA, we sought to investigate the relationship between CYT, ICG expression, and survival in patients with GBM. Studies performed in other cancer types have indicated a survival benefit in patients with increased CD8 T cell infiltration and CYT^4,6^. The literature in GBM, however, is mixed and conflicting at times. For example, while both Kmiecik et al*.* and Lohr et al*.* found improved GBM patient survival with increased T cell infiltration, Ndoum et al*.* found that patients with higher CYT had reduced survival, and Martinez-Lage et al*.* found no relationship between T cell infiltration and survival^8,9,11,19^. Ndoum et al. also highlighted a positive relationship between CYT and PD1/PDL1 expression^10^. Beyond PD1/PDL1, however, there has yet to be literature investigating how CYT and other ICGs are related to survival and immune cell infiltration in GBM, thus motivating our study.

In our analysis of the multi-institutional TCGA database, we found that patients with higher CYT had significantly reduced survival, as previously reported^10^, and that this relationship remained significant on multivariate analysis (Table 2). This is in contrast to trends seen in other cancer types^4,6^ and highlights the unique relationship between GBM and the immune system. In theory, higher CYT would imply greater infiltration of tumors with immune cells that can induce an antitumor response. However, the reduced survival observed despite higher CYT in GBM perhaps reflects the profound immunosuppressive microenvironment and T cell dysfunction that is characteristic of this malignant tumor^13^. Studies in non-small cell lung cancer and renal cell carcinoma have demonstrated a positive correlation between CD8+ T cell infiltration and tumor grade, potentially related to the increased number of tumor-associated neo-antigens in dedifferentiated cancers^20,21^, A similar mechanism may be occurring in GBM, as the mesenchymal subtype of GBM, which carries the worst prognosis, and hypermutated tumors are both associated with increased lymphocyte infiltration^11,14^. GBM has a very low tumor mutation burden leading to a reduced number of neo-antigens compared to other cancers, suggesting that mutational status may not be the sole reason for increased T cell infiltration^22^. An alternative pathway involves recruitment of lymphocytes to GBM via a mechanism associated with more aggressive biology and increased disruption of the blood brain barrier, allowing for increased tumor immune cell infiltration rather than as a reaction to tumor-associated neo-antigens. ICG expression may also contribute to the reduced survival seen in patients with high CYT scores, given the strong correlation between ICG and CYT (Fig. 3). Many of the ICGs in our study, including IDO, PDL1, PD1, TIM3, and CTLA4 expression have been directly associated with reduced survival in GBM patients, while LAG3 has been tied to severe T cell dysfunction^10,13,14,23–25^.

As expected, patients with HighCYT/HighICG had reduced survival relative to LowCYT/LowICG patients across all individual ICGs and the final composite ICGscore as well (Fig. 2). The correlation of CYT with ICG expression perhaps relates to the tumor microenvironment inappropriately utilizing endogenous immune negative feedback loops. In cancers such as melanoma, similar trends have been observed, with increased CD8+ T cell infiltration being associated with increased IDO, PDL1, and Treg levels in an IFN-gamma-dependent manner^26^. This correlation also likely contributes to the low number of patients in HighCYT/LowICG subgroups across the various ICGs, limiting conclusions that can be drawn regarding that rare subgroups of patients. Similarly to the trends seen in melanoma, increased IDO expression has been tied to CD8+ infiltration and IFN-gamma expression in GBM^12^. Other ICGs included our analysis, including PD1, TIM3, CTLA4, and LAG3 are well known markers of T cell exhaustion^13,27^. Woroniecka et al. has demonstrated increased levels of PD1+ T cells within GBM patient tumor samples with coexpression of TIM3, LAG3, or both, indicating a severely dysfunctional T cell phenotype^15^. This may also explain the relatively modest, although still significant, direct relationship between LAG3 and CYT, as severely dysfunctional T cells are frequently double positive for LAG3 and PD1. Woroniecka et al. also demonstrated that the majority of CD8+ T cells within human GBM were of the effector memory T-cell phenotype^15^. Thus, it seems that although some patients may have T cells recruited to the tumor and initially activated, they then become exhausted in the severely immunosuppressive GBM microenvironment. This is further supported by our findings that correlate CYT, which is representative of T cell infiltration and activation, with ICG expression.

HighCYT/HighICGscore patients also had higher levels of tumor-promoting and immunosuppressive M2 Macrophages and Neutrophils^18,28–31^, with lower levels of potentially anti-tumor resting NK cells. These trends are also in contrast to other cancer types such as colorectal cancer, where CYT is correlated with anti-tumor M1 Macrophage infiltration^4^. This may be related to the significantly higher number of mesenchymal tumors seen in the high CYT group of patients, as a mesenchymal tumor phenotype is associated with increased M2 macrophage infiltration^11,23^. GBM associated macrophages have also been shown to express PDL1^32,33^, potentially contributing to the correlation between CYT and PDL1 expression demonstrated in our findings. M2 macrophages also contribute to the immunosuppresive microenvironment of the tumor through the expression of immunosuppressive signaling molecules, such as IL-10 and TGF-ß^33^. Similarly to macrophages, increased neutrophil infiltration into solid tumors has also been associated with a mesenchymal subtype, VEGF resistance, and a worse prognosis in patients with GBM^18,28,29^. As a result, the increased infiltration of pro-tumoral myeloid cells may contribute to the reduced survival seen in the HighCYT/HighICGscore subgroup. Another important consideration is that the CIBERSORT program used to determine immune cell infiltration does not identify myeloid derived suppressor cells (MDSCs), potentially resulting in the mislabeling of pro-tumoral polymorphonuclear MDSCs, which have also been associated with reduced survival in GBM^34^, as neutrophils given the overlap in gene expression between the two. Additional investigation into the association between T cell and myeloid cell infiltration utilizing flow cytometry and immunohistochemistry in unison with RNA sequencing data could verify the trends described in this study.

To further validate the findings described above, we next applied the same methodology to a cohort of GBM patients from the CGGA. Findings using the CGGA database were similar to those from TCGA, with significantly reduced survival in patients with HighCYT vs. LowCYT. Subgroup analysis also displayed similar trends to those seen when utilizing TCGA with LowCYT/LowICGscore patients trending towards improved survival when compared to HighCYT/HighICGscore patients, albeit not reaching statistical significance (p = 0.066). In addition, ICGscore strongly correlated with CYT in the CGGA database, corresponding with TCGA findings. Comparable trends were also seen with regards to immune cell infiltration, including immunosuppressive M2 macrophages. However, differences seen in neutrophil and NK cell infiltration were abrogated when utilizing the CGGA database. Differences in findings between the two cohorts may be due to underlying ethnic differences and differences in tumor physiology in TCGA vs. CGGA. Indeed, patient survival in the CGGA cohort was over double the survival seen in the TCGA database, suggesting differences in underlying tumor physiology and, potentially, classifications of tumor grade. While promising that the majority of trends seen in the TCGA cohort were also seen in the CGGA patient cohort, further investigations into the findings described in this paper may benefit from the utilization of a larger, more uniform, database with downstream verification of transcriptomic findings.

The limitations of our study are primarily related to its retrospective nature and the underlying bioinformatics approach utilized, limiting the mechanistic conclusions that can be drawn. In addition, the study primarily relies on the use of transcriptomic data, without validating findings through additional cell phenotyping modalities (e.g., flow cytometry or immunohistochemistry). Further investigations into the relationship between GBM T cell infiltration, patient survival, and local immune suppression, including immunosuppressive gene expression and immune cell infiltration, utilizing these methodologies are needed.

This study highlights a complex immunosuppressive tumor microenvironment with increased ICG expression and pro-tumoral immune cell infiltration in GBM patients with increased CD8+ T cell infiltration and activity, as indicated by the CYT. As their tumors are already inflamed with CD8+ T cells that seem to be held in check by multiple aspects of the tumor microenvironment, patients with HighCYT/HighICGscore may respond particularly well to immunotherapies, such as immune checkpoint blockade, that would remove the immunosuppressive signals hindering the CD8+ T cells from realizing their purpose. Further research is required to fully elucidate the relationship between CD8+ infiltration and immunosuppression in the GBM tumor microenvironment, especially any potential relationship between CD8 infiltration and pro-tumoral myeloid cell infiltration. Potential future studies could assess how CYT and ICGscore phenotypes potentially predict treatment responses in GBM patients receiving immunotherapies.
